# Periprosthetic fractures of the knee: a comprehensive review

**DOI:** 10.1007/s00590-019-02582-5

**Published:** 2019-11-19

**Authors:** Vadim Benkovich, Yuri Klassov, Boris Mazilis, Shlomo Bloom

**Affiliations:** 1 Department of Joint Arthroplasty, “Yonatan” Center-Israeli Joint and Spine Health Center, Assuta Medical Center, Soroka University Medical Center, Beer-Sheva, Israel; 2 Orthopedic Surgery Department, Soroka University Medical Center, Beer-Sheva, Israel; 3 Ben Gurion University, Soroka medical Center, Beer-Sheva, Israel

**Keywords:** Femur, Tibia, Periprosthetic fracture, Knee, Arthroplasty

## Abstract

Demographic changes have resulted in an increase in the number of older patients diagnosed with degenerative joint disease. Developments in the field of joint arthroplasty allow a broader population to improve their lifestyles. An increased demand for knee arthroplasty has led to a rise in operations performed worldwide. Although there has been a constant propagation of technology and an increase in medical staffing at a professional level, many patients still encounter complications. Though rare, these factors may lead to life-threatening scenarios and a devastating effect on the success of the operation. One such rare complication includes periprosthetic fractures around the knee, a complex injury which requires a cautious and experienced approach. In this review, we analyze the prevalence, risk factors and classification, investigation and treatment options for periprosthetic fractures with total knee arthroplasty.

## Introduction

In 2012, 700,100 total knee replacements were performed in the USA. This was the single most frequent procedure completed during hospital stays among patients. In addition, knee arthroplasty was the second leading procedure with the greatest change in occurrence from 2003 to 2012. The rate of knee arthroplasty grew by more than 50% over this time when measured in a population of 100,000. The incidence of knee arthroplasty increased from 145 to 223 during that time period, with an annual rate of change of 4.9%. When looking at the distribution of those procedures among different age groups, 663,600 procedures were performed in patients aged 65–84, which accounts for 94.7% of the total procedures. Regarding the gender of those undergoing knee arthroplasty, women constituted 60% of patients and males made up 40% of those requiring the procedure [1]. Regarding this data, we can understand that due to a tremendous rise in the number of total knee arthroplasties (TKR/TKA), it is expected that the incidence of periprosthetic fractures around the knee will rise accordingly.

Treatment of complications is a complex and resource-consuming task. Why is it important to discuss these complications? Firstly, the economic burden from revision TKA’s performed in the USA has risen to a tremendous $2.7 billion. This number is expected to increase as the procedure rate increases as well [2]. In a retrospective study conducted by Streubel [3] regarding mortality associated with periprosthetic fractures of the femur, it was reported that patients with periprosthetic femur fractures have a similar or higher mortality risk than hip fracture patients. In addition, periprosthetic femur fractures carry a significantly higher mortality risk than either hip or knee arthroplasty or operative fixation of native distal femur fractures. In accordance with that, the 30-day, 6-month and 1-year mortality rates of patients with periprosthetic distal femur fractures were 8, 24, and 27%, respectively [4].

This review article presents and analyzes the incidence and prevalence of these fractures, various risk factors, proposed classification systems and current treatment options. The article focuses mainly on femoral periprosthetic fractures due to their higher prevalence.

## Epidemiology

Delanois et al. [2] assessed the incidence of periprosthetic fractures and revision operations performed using the National Inpatient Sample (NIS). It was found that between 2009 and 2013, 337,597 patients had a revision TKA procedure performed. Among those patients, the mean patient age was 65 years, with 60% of patients aged 64–74. In addition, women accounted for 58% of the patients. The most common etiologies for revision TKA included infection and mechanical loosening. Of all the diagnosed reasons for a revision procedure, periprosthetic fracture was the least reported. In addition, we analyzed data from the Italian Arthroplasty Registry Project [5] which reported a total of 181,738 joint replacement procedures performed in 2015, a rise of 3.7% compared to procedures performed in 2014. Regarding revision TKA procedures, aseptic loosening was the prevailing cause in 33.3% of cases, while infection was the cause in 27% of cases. However, periprosthetic fracture accounted for a mere 1%.

Among periprosthetic fractures, femoral supracondylar periprosthetic fractures are the most common with an incidence rate of 0.3–2.5% [6] post-primary TKR. However, those numbers can rise up to 38% [7] in cases of revision surgeries. Periprosthetic fractures of the tibia occur at an incidence of approximately 0.4–1.7% in primary TKR and approximately 0.9% in revision TKR. Periprosthetic fractures of the patella occur with an incidence of 0.2–21% of cases, depending on the eventual patellar resurfacing, which can increase the incidence [8]. We believe that those numbers may be underestimated due to the fact that many of those fractures are treated conservatively, or with open reduction internal fixation (ORIF), and are not reported.

## Risk factors

Predisposing factors can contribute to the development of periprosthetic fractures. The most common, advanced age, is emphasized in this work. Advanced age is a major risk factor regarded as an individual risk factor within itself, as well as a risk factor for osteoporosis and recurrent falls, each of which on their own are qualified as risk factors for periprosthetic fractures. An interesting debate has arisen regarding the relationship between age and the risk of periprosthetic fractures. In an article by Meek et al. [9], it was reported that females older than 70 years were at an increased risk of periprosthetic fractures. In contrast, in an article by Singh et al. [10], it was concluded that patients at an age ≤ 60 years were associated with a higher risk of postoperative periprosthetic fractures following primary TKR.

Additional risk factors include the chronic use of steroid therapy, inflammatory arthropathy such as rheumatoid arthritis, and patients suffering from neurological diseases including epilepsy, Parkinson’s disease, poliomyelitis, and myasthenia gravis which all appear to increase the risk of periprosthetic fractures [8, 11]. Diabetes mellitus (DM) is an additional risk factor that has an important effect. DM may affect the stability of patients and, therefore, contributes to the known risk factor—recurrent falls [12]. It can also affect the post-surgical healing process due to microvascular and neural damage [11]. A further important risk factor that can influence the integrity of the prosthesis is obesity [13]. Today, it is well reported that obese people have impaired functionality and mechanical outcomes following TKA.

Revision TKA was in itself described as another major risk factor for the development of periprosthetic fractures. In a large population-based study conducted by Meek et al. [9] from 2011, 44,511 primary TKA and 3222 revision TKA procedures were performed. The authors reported that the risk of fracture after primary TKA was 0.6% versus 1.7% after revision TKA. As this research is a large reliable population study, it provides a good prospective regarding the true numbers surrounding periprosthetic fractures in the last years. An additional study conducted by Singh et al. [10] reviewed 12,914 patients who underwent 17,633 primary TKRs and 3286 patients who underwent 4090 revision TKRs during the period of 1989–2008. The researchers concluded that 1.1% of patients after primary TKR and 2.5% of patients after revision TKR sustained a postoperative periprosthetic fracture on, or after day one, postoperative. Along with the findings regarding revision surgeries as well as the increased incidence of periprosthetic fractures, Singh et al. concluded that in patients with revision TKR, diagnosis of nonunion, infection and previous surgery with components removed were significant predictors of postoperative periprosthetic fractures. Comparing patients with loosening, wear and osteolysis, those with previous nonunion were almost 5 times more likely to suffer from a postoperative periprosthetic fracture.

Another important, yet controversial, factor to discuss is femoral notching. After reviewing the literature pertaining to the subject, the prevalence of anterior cortex notching of the femur is estimated at 3.5–41% [14–16]. A previous study reviewed the biochemical analysis of fresh-frozen cadaveric femora. It was found that torsional strength testing had a 31% decrease in distal femoral torsional load to failure following femoral notching. Furthermore, it presented an 18% decrease in bending strength, and a mean reduction by 39.2% in torsional strength of the anterior femoral cortex [17]. However, when reviewing three papers written by Ritter [14], Minarro et al. [15], and Gujarathi et al. [16], results were contradictory. Ritter et al. and Gujarathi et al. analyzed the incidence of femoral notching and the presence of supracondylar femoral fractures, with a long follow-up period between 5 and 9 years. Both authors concluded that there is no relationship between minimal anterior femoral notching and supracondylar fracture of the femur. Minarro et al. [15] studied the fracture pattern relation with the anterior femoral notching. The author concluded that the fracture pattern is not related to the existence of a femoral notch in the clinical setting. Our conclusion is that in the clinical field, the occurrence of fractures due to notching is very small or nonexistent.

## Classification

Throughout orthopedic medicine, there are many classifications with respect to every subject and fracture. In regard to the femur, there are several classification systems for femoral supracondylar periprosthetic fractures including: Neer and Associates [18] , DiGioia and Rubash [19], Chen and Associates Classification [20] and Su and Associates Classification of supracondylar fractures of the distal femur. An additional classification we would like to address is the Unified Classification System (UCS) [17]. This classification has been chosen by the AO (“Association for the study of internal fixation”) as the primary classification for periprosthetic fractures. In this classification, there are six different classes of fractures, categorized from A–F. Each category describes a specific anatomical description of a periprosthetic fracture. All of the classes can be utilized in different anatomical locations, as long as one follows the classification principles. In an article from 2014, written by Duncan and Haddad [21], the author presents the use of the UCS as a practical tool that can be applied on every anatomical part in the musculoskeletal system, regardless of the exact location of the periprosthetic fracture. Nevertheless, the most widely used classification today, to our knowledge, is Rorabeck and Tylor [22], which considers both fracture displacement and prosthesis condition [23, 24]. We agree with the basic hypothesis that most, if not all, periprosthetic fractures can be classified by one unanimous classification system that promotes a rational approach to treatment. In our clinical practice, we do so by introducing the UCS system to all of our orthopedic residents as a classification system that can exhibit the correct approach to all periprosthetic fractures. It is the opinion of this study that the reason for these numerous approaches to classification grows from an inability to find a proper classification system that will cover every question asked by the orthopedic surgeon.

**Fig. 1:**
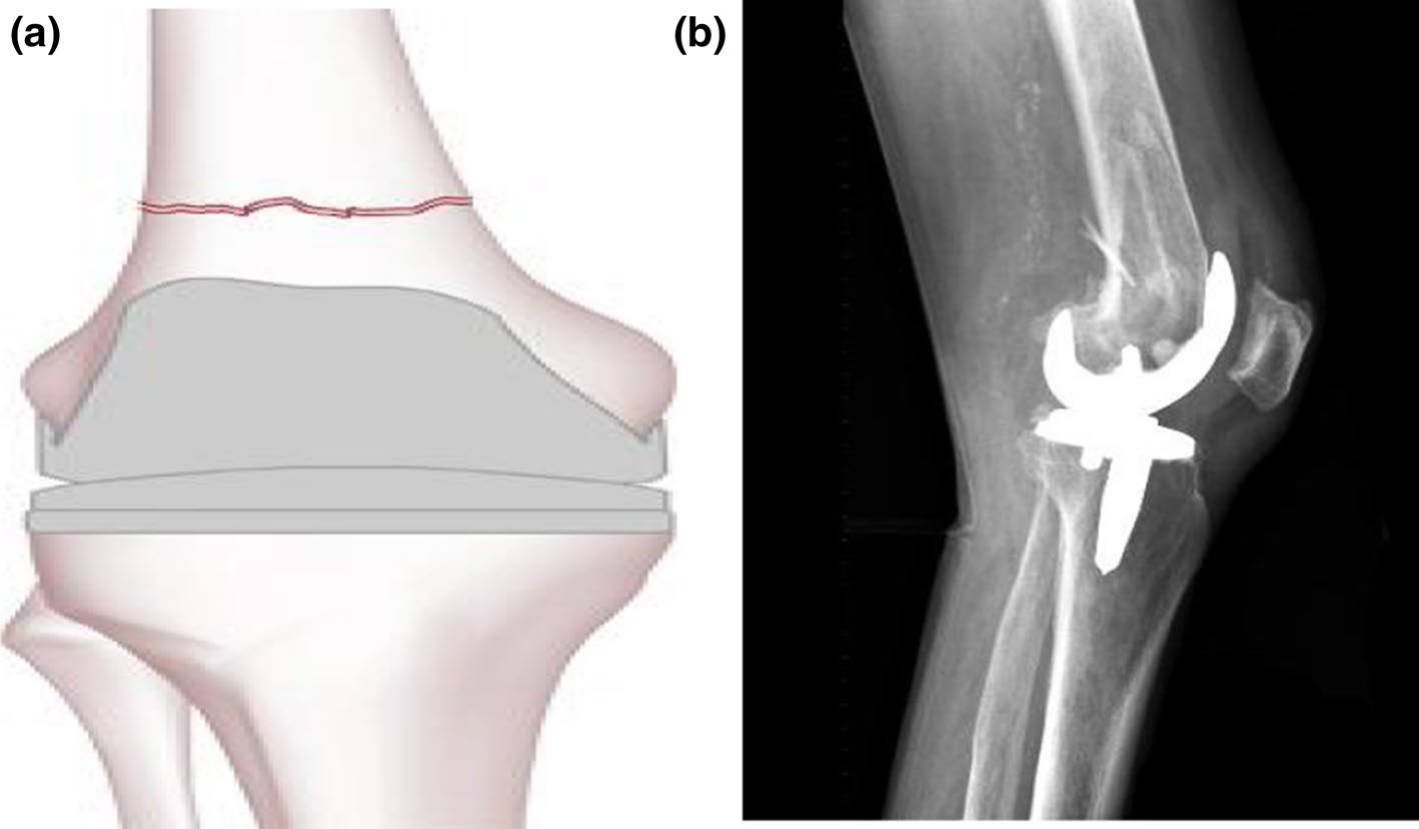
Type I Rorabeck and Tylor: a non-displaced fracture and the prosthesis is intact

**Fig. 2:**
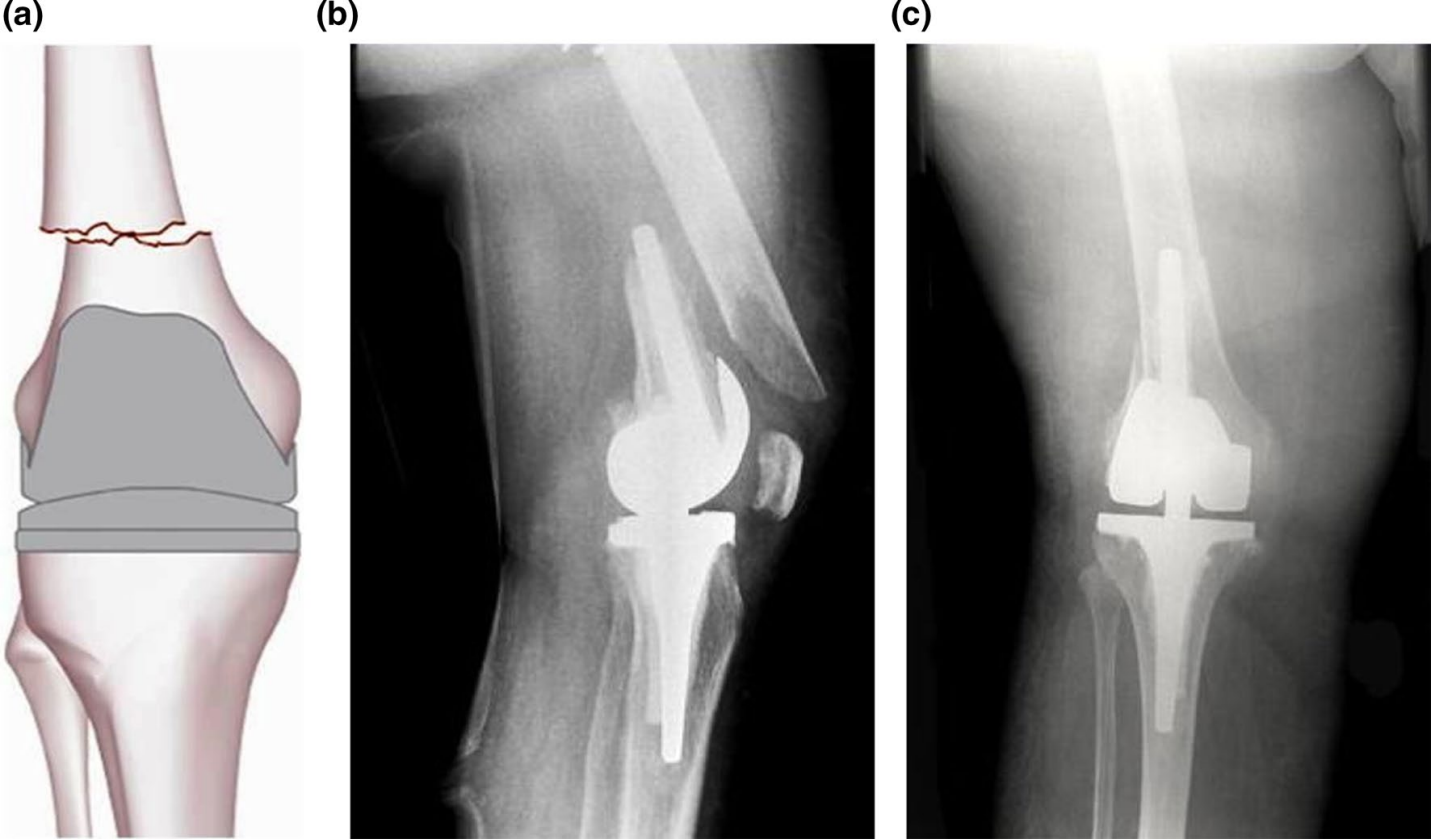
Type II Rorabeck and Tylor: a displaced fracture and the prosthesis is intact

**Fig. 3:**
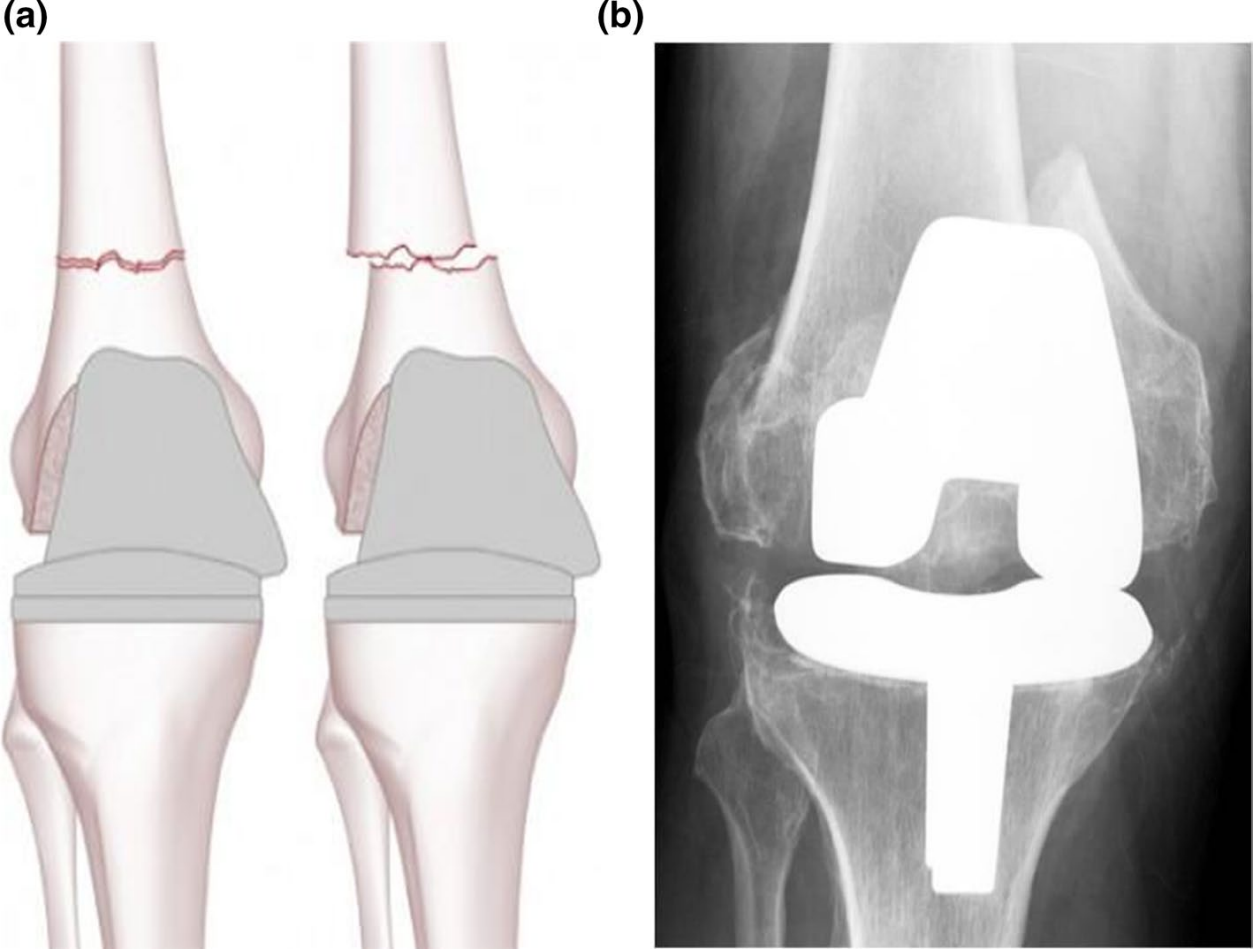
Type III Rorabeck and Tylor: a non-displaced or displaced fracture. The prosthesis is loose or failing

**Fig. 4:**
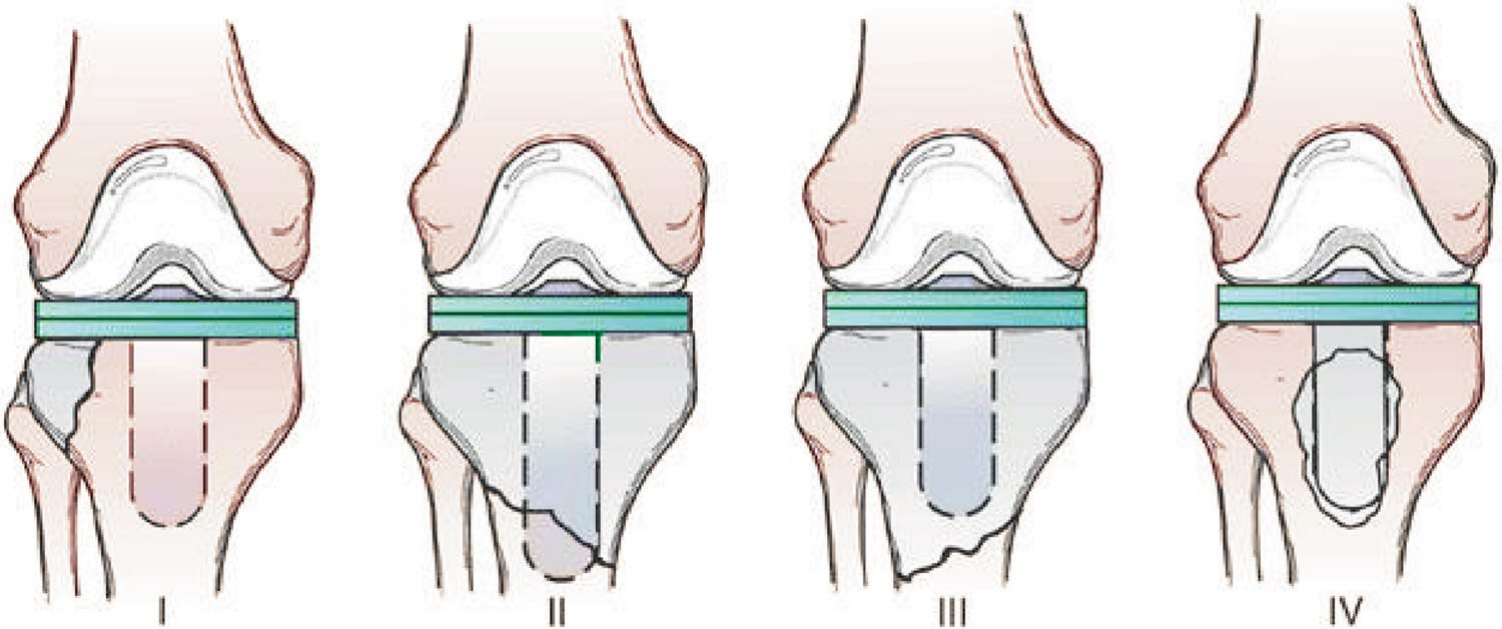
Felix and associates tibial periprosthetic fracture classification

Rorabeck and Tylor Types I, II and III (Figs. 1, 2, 3):

Although tibial periprosthetic fractures occur less frequently than femoral fractures, we would like to present a classification system that is used for tibial periprosthetic fractures. This classification was introduced in 1997 by Felix and associates [25], who based their classification on 102 periprosthetic tibial fractures. The model set by Felix and associates is used most commonly because it provides a guide for determining the appropriate treatment for tibial fractures associated with total knee arthroplasty [26]. It is classified as follows (Fig. 4):

- Type I: Fracture of tibial plateau
- Type II: Fracture adjacent to tibial stem
- Type III: Fracture of tibial shaft, distal to component
- Type IV: Fracture of tibial tubercle

**Fig. 5:**
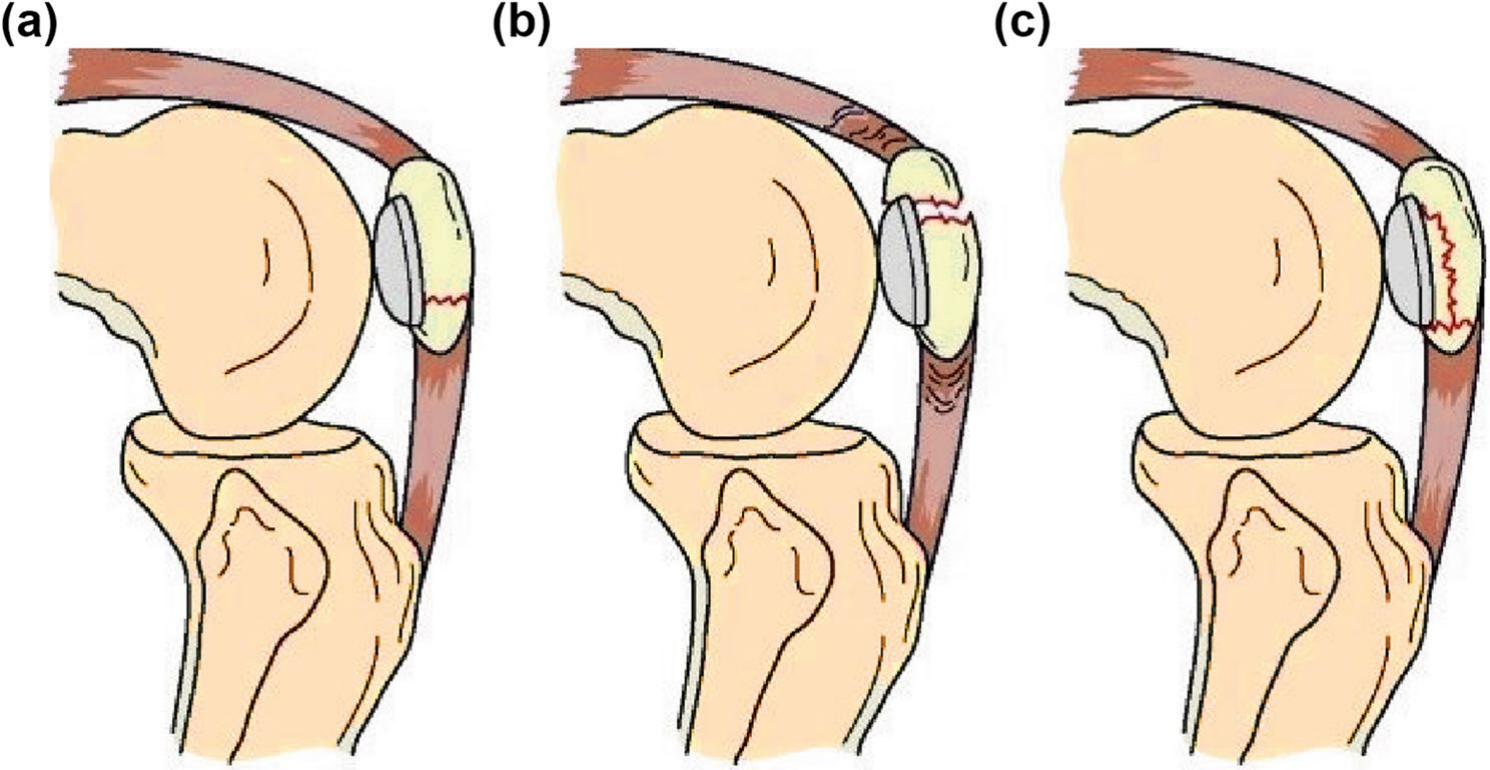
Patellar periprosthetic fractures classification

Patellar periprosthetic fractures are classified in a similar way to tibial and femoral fractures while taking into account both component stability and quality of the bone, together with integrity of the extensor mechanism (Fig. 5) [27].

- Type I is most common and is frequently asymptomatic and discovered on routine follow-up radiographs. These fractures present with a well-fixed implant and an intact extensor mechanism.
- Type II is associated with disruption of the extensor mechanism, but retains a well-fixed implant. This type is associated with a high rate of complications (50%) and recurrent operations (42%).
- Type III is associated with a loose patellar implant, and is divided further, as related to bone stock: 3a is implemented if there is a good remaining bone stock, while 3b is implemented if the remaining bone stock is of poor quality.

## Evaluation and analysis

When a patient first comes to the emergency room, it is pertinent that the physician acquires a history about his or her comorbidities, chronic medications, trauma and whether or not he or she has suffered from pain associated with the joint before fracture occurred. This is critical as it may suggest that there was a pre-existing aseptic loosening of the implant, possibly due to component loosening, polyethylene wear with osteolysis, ligamentous laxity, arthrofibrosis or patellofemoral complications [28]. Loosening due to infection could occur as well, and begins with a history of pain, swelling, erythema and prolonged wound drainage or a sinus tract [29]. In such a case, we suggest acquiring a white blood cell count, erythrocyte sedimentation rate and C-reactive protein. If these counts are raised, the next step should involve aspiration of the joint for cell count and differential, done along with culturing. In addition, a meticulous physical exam should be performed. Following the history and physical exam, physicians should move forward to standard anteroposterior (AP) and lateral radiographs of the knee views, as they are the basis of fracture analysis and classification. Typical signs of loosening will appear as displacement of the femoral component or stem with a complete radiolucent line of 2 mm or more around the prosthesis at the bone–cement interface. In more complex cases, where the fracture pattern cannot be completely identified with AP and lateral X-rays, a CT-scan may aid in the detection of loosening or to gain a clear picture of the fracture pattern. If previous radiographs are available, they should be analyzed for comparison [30, 31].

## Management

**Fig. 6:**
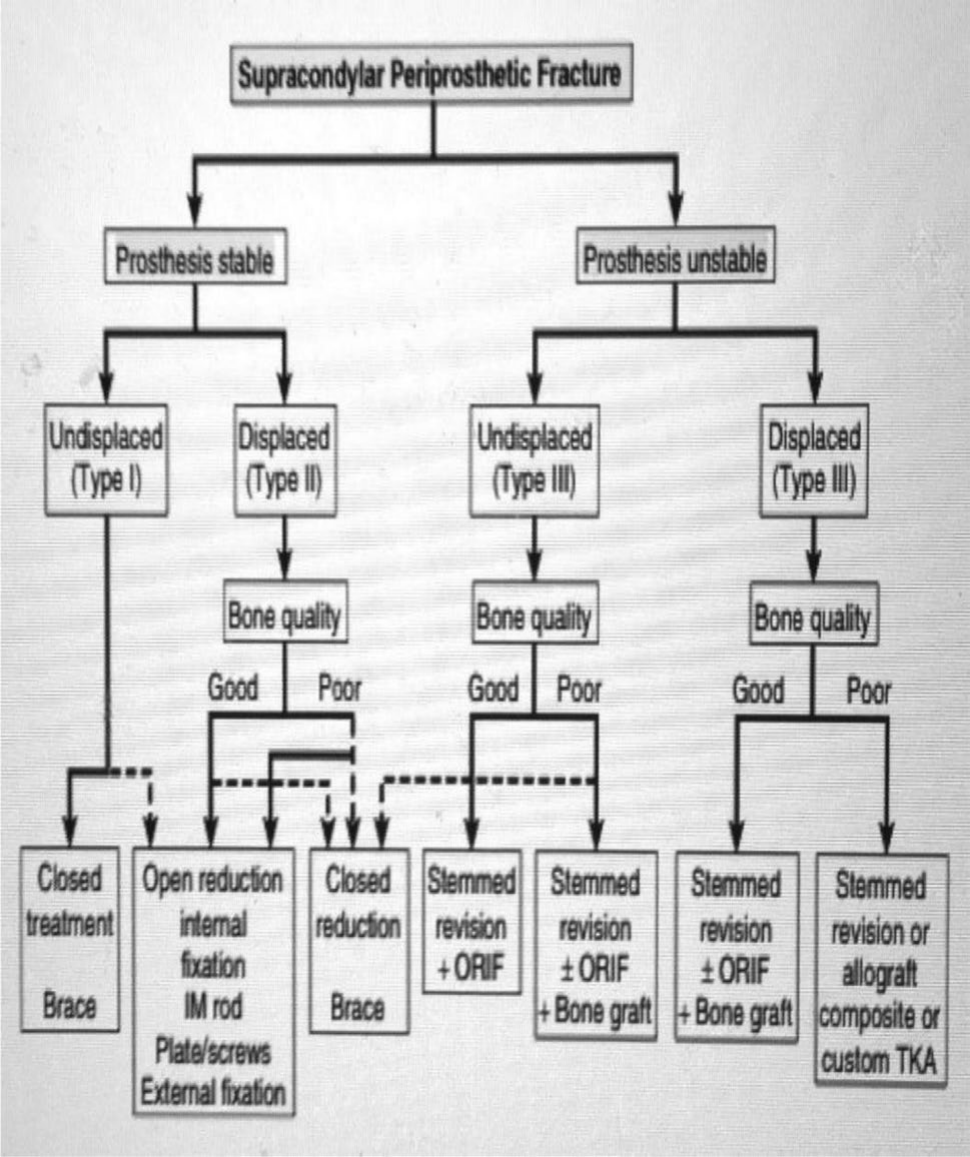
Treatment algorithm for supracondylar periprosthetic fractures. IM, intramedullary rod; ORIF, open reduction and internal fixation; TKA, total knee arthroplasty

Treatment of periprosthetic fractures should aim to achieve a painless and stable knee with proper restoration of alignment, adequate patellofemoral function with maintenance of prosthesis fixation, and early range of motion that allows the patient to return to his or her usual life style with the same capabilities prior to the fracture, as soon as possible. Regarding femoral, tibial, and patellar fractures, treatment algorithms exist which are simple to understand, applicable to daily life, and are based on the classification systems that are used most commonly. In the Rorabeck and Tylor treatment algorithm (Fig. 6), the non-displaced, stable fractures with well-fixed implants are treated non-operatively, while displaced fractures with well-fixed implants are treated with internal fixation. Unstable prosthesis, with good or poor bone quality, is always treated with revision of previous prosthesis, with or without bone grafting.

**Fig. 7:**
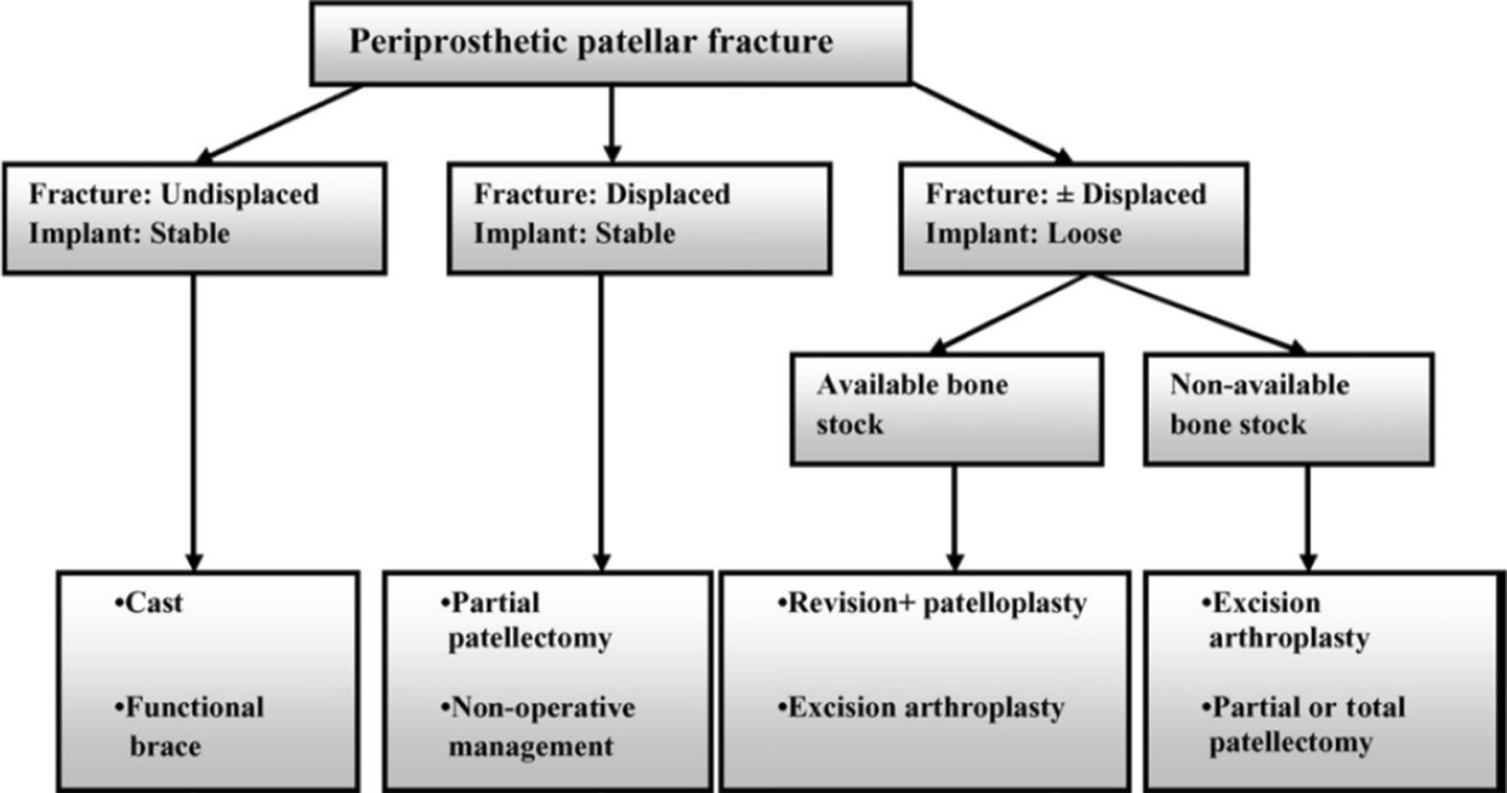
Treatment algorithm for patellar periprosthetic fractures

In a work by Ortiguera and Berry [32], researchers proposed a treatment algorithm for patellar periprosthetic fractures which is based upon the stability of the implant and whether the extensor mechanism is intact or disrupted. An additional management of a post-TKA patellar fracture was described by Chalidis et al. [33] (Fig. 7). There, it is proposed that if the extensor mechanism and the implant are intact, then the treatment is non-operative with good results [34, 35]. If there is a stable implant but a disrupted extensor mechanism, the fracture involves one of the poles of the patella. To avoid fragmentation of the residual bone, it is advised to leave the implant in place and reconstruct the extensor apparatus [36]. If there is a more severe fracture that involves loosening of the implant, then the condition of the bone stock should be examined. Whether the bone stock is good or poor determines whether one should perform patelloplasty with component revision or a complete patellectomy.

Tibial periprosthetic fractures are uncommon; nonetheless, they should be discussed. The treatment algorithm that we use is based upon that described by Felix et al. [25, 37]. Using this algorithm, classification separates fractures on the basis of location, implant stability, and timing. Fractures that are associated with loose implants are treated with revision, bone grafting, and stemmed implants. However, if the fracture is not displaced and is stable with well-fixed implants, then the treatment rationale is to handle it non-operatively. A fracture that is displaced but retains a stable implant should be treated with internal fixation. If the implant is unstable, then revision endoprosthesis should be performed.

## Intraoperative and postoperative fractures

Intraoperative fractures are said to be relatively easy to manage as they are usually non-displaced and the implant remains stable, along with the fact that there is not much of soft tissue compromised [38]. Femoral fractures around the metaphysis are usually managed with a single trans-condylar screw, and a diaphysis fracture can be fixated or stabilized with a stem that will pass the perforation by two or more cortices dimensions [23]. Tibial fractures occur more commonly during revision than primary total knee replacement [39]; however, they are less common than femoral fractures [38]. The treatment tactics for tibial intraoperative fractures are similar to those used regarding femoral. Patellar fractures that will occur in the operating setting will most likely be non-displaced and may be treated conservatively or by tension band wiring procedure if there is concern for the extensor mechanism integrity. These are also considered quite rare complications. All of those cases will most likely require a period of protected weight-bearing and monitoring.

Postoperative periprosthetic fractures around the knee should, in our opinion, be followed with the algorithms that we presented in this article as they are based on the most widely used classification systems [22–24, 26, 27, 30, 32–34].

In the following paragraphs, this review will present and discuss various treatment options.

## Non-operative treatment

Non-operative treatment is an important component of orthopedic surgery. The operative approach has its complications, which include anesthesia and surgery that can cause postoperative infections, excessive bleeding, failure of fixation devices, etc. The concept of non-operative treatment involves cast application with or without a traction period.

There are several studies that report satisfactory results with non-operative treatment [34]. In a large review written by Chalidis et al. [33], they compiled 19 papers with data regarding the treatment of periprosthetic patellar fractures. Of those, 67% were treated non-operatively with observation or cast application, of which all reported positive results. The purpose of a study written by Agarwal et al. [40] was to evaluate the outcome in patients treated for periprosthetic fractures. Out of 15 patients with supracondylar femoral fractures, 2 were Roraback and Lewis Type I (non-displaced, with intact prosthesis–bone interface). They were treated with immobilization in a long leg cast. Follow-up occurred with patients over 24 and 34 months. Both patients presented excellent results in the range of motion, knee score, and functional score. In an article by Merkell and Johnson [41] from 1986, researchers reviewed the data on 36 supracondylar fractures of the femur. Of those, 26 fractures had been treated using non-operative methods. Seventeen of those fractures (65.4%) healed without surgical treatment. Fourteen of the 17 were followed for over 2 years and did not present with significant differences in the knee score over this time. The remaining patients required knee revision surgery due to nonunion, malunion, loosening of the component, and extension lag. Nonetheless, they concluded that traction or application of a cast, or both, should be the primary treatment options, and usually one will result in healing of the fracture and a satisfactory outcome.

We do acknowledge that this article might look obsolete. However, the basic principles of treating a non-displaced fracture in an elderly patient in whom the surgical risk is high are a reasonable decision.

In summary, we claim that conservative treatment should be used if the fracture is non-displaced and the component is stable. If the patient is not eligible for operation or if the risk of the operation is very high, this can only strengthen the reason to use conservative treatment. The use of non-operative techniques demands that those patients are examined closely, followed by routine imaging in order to determine that there is satisfactory alignment and fracture healing. Surgical intervention can always be considered until full healing occurs.

## Operative treatment

There are many operative methods from which to choose in regard to treatment. Here, this review will discuss the most widely used current fixation methods. The type of fixation method depends largely on how well the implant is fixed to the bone, what the fracture pattern is, whether or not there is an active infection process, the quality of the bone, and whether there is a sufficient amount of bone stock or if augments must be used. Just as important are the skill set of the operating surgeon and what type of equipment is provided by the hospital in order to perform the surgery. Our goals as orthopedic surgeons dealing with periprosthetic fractures are to achieve satisfactory fixation and to restore proper alignment, with as much preservation as possible of soft tissue while preventing intraoperative and postoperative complications.

## Plate fixation

Open reduction and internal fixation allow the surgeon to perform anatomical reconstruction, enabling the patient to perform early rehabilitation. As in every aspect of surgery, there are those who oppose and those who support this fixation technique. We can divide plates into types: non-locking (conventional) plating and locking plates. We can further divide locking plates into the following categories: variable-angle or fixed-angle screw orientation.

In an article written by Hassan et al. [42], researchers reviewed a hospital database of 26 patients with periprosthetic fractures after total knee arthroplasty. Researchers concluded that the use of locking plates had a 96% union rate, yet complete healing took 6 months and full weight-bearing was possible in 94% of patient at 3 months.

Hoffmann et al. [43] retrospectively reviewed 111 fractures in 106 patients who underwent locked plate fixation due to periprosthetic fractures around the knee. Thirty-six fractures were treated with the open reduction method, and 75 fractures were treated with minimally invasive submuscular plate application. Of the total number of fractures, 91% healed completely. Interestingly, the research found that there was a decreased frequency of nonunion among those whose fractures were treated by the minimally invasive submuscular technique compared to those treated with the open technique.

The primary objective of a study written by Alexander et al. [44] was to compare biomechanical failure properties of three proximal plate fixation techniques (bicortical locking, unicortical locking, and cerclage cable configuration) in a periprosthetic distal femur fracture in an osteoporotic bone model. A segmental defect was created in 21 synthetic osteoporotic adult femurs. All specimens were stabilized with a 246-mm locking femur plate. Fixation in the most proximal hole was varied by use of either a cerclage cable, unicortical locking screw, or a bicortical locking screw. The distal fixation of the plate was the same for all the specimens. Proximal cerclage fixation demonstrated higher mean maximum axial force at failure, stiffness, and maximum torque.

We can see that fracture fixation with plating can be achieved with open reduction when the fracture is under direct vision of the surgeon, a process that is very comfortable for fixation and very helpful in cases that involve a complex fracture, or when the surgeon does not have the proper amount of experience. This has worse nonunion rates when compared with minimally invasive techniques, but still yields very good results. We can conclude that, in our opinion, with regard to plating, the “mini open” technique with cerclage wiring and the use of a polyaxial locking plate is the preferable technique today with regard to soft tissue preservation, but requires an experienced surgeon and is more difficult to perform in complex fractures.

## Retrograde intramedullary nailing

There is a dispute regarding intramedullary nailing versus open reduction and internal fixation with plating. When comparing nailing to non-locked plating, we can see by reviewing articles which examined these fixation methods that intramedullary nailing retains an advantage in operative time and intraoperative blood loss with a relative risk reduction of 87% for developing a nonunion and 70% for requiring revision surgery.

Currently, we commonly use locking plates, so we will present here articles that compare intramedullary nailing and ORIF made with locking plates. In an article by Meneghini et al. [45], researchers analyzed the outcomes of intramedullary (IM) nails with a locked distal screw versus periarticular locking plates. Eighty-five fractures were reviewed between 2001 and 2011. All fractures in their study were Lewis and Rorabeck Type II. Fixation was performed in 22 knees with a retrograde IM nail and in 63 periarticular locked plates. Researchers concluded that the IM nailing group had only 2 nonunions when compared with the locking plate group, which experienced 12 nonunions or malunions. Furthermore, the IM nailing group achieved full weight-bearing ambulation post-op at 9.1 weeks, while the locking plate group achieved the same by 11.7 weeks.

Contrary to the previous article by Meneghini et al., one large systematic review written by Ristevski et al. [46], which included a total of 719 fractures, showed a clear advantage of locked plating over intramedullary nailing when comparing malunion rates. Authors attribute this to the difficulty in obtaining the correct starting point as dictated by the femoral component (thus achieving a precise reduction is more difficult) in addition to the difficulty filling the wide metaphyseal flare and fewer distal fixation options for fractures distal to the anterior flange of the femoral component (thus, making it even more difficult to maintain a reduction).

Contrary to both of those articles, Kilicoglu et al. [47] found no differences regarding alignment, Knee Society score, range of motion, or time to union between the retrograde intramedullary nailing group as compared to a group treated with locked plating. In articles by Gliatis et al. [48], Han et al. [49], and Chettiar et al. [50], all showed that periprosthetic supracondylar distal femur fractures treated with retrograde IM nailing collectively reported a 100% union rate, with only 1 reported fracture healing in malalignment. Collectively, these studies examined 32 patients. The problem with these articles is that each reviews only a small number of participants, and there are no comparisons to other fixation methods.

In addition, the application of poller, blocking screws, or pins is a useful technique utilized to improve the reduction and the final alignment of the femur [51]. Taken together with this technique, it was found that an increased number of distal interlocking screws were found to have reduced the risk of nonunion and reoperation rates [52]. Intramedullary nailing proved itself a good fixation technique for periprosthetic fracture fixation; there is no absolute proof which is better, locking plates or intramedullary nailing, but there are issues that we want to discuss solely for those who choose to use intramedullary nailing.

One such issue that has to be considered in preoperative planning is the type of the total knee prosthesis components and whether the intercondylar notch can be accessed in order to provide a safe passage for the nail. In an article written by Service et al. [53], researchers evaluated the influence of distal femoral prosthetic design at the starting point of retrograde nail insertion. They examined 100 lateral knee images and analyzed femoral components from six manufacturers. They concluded that many femoral component designs, especially cruciate retaining, pose the risk of placing the starting point of a retrograde nail posterior to Blumensaat’s line, hence predisposing it to recurvatum deformity and malalignment.

Luckily for us, in an article by Thompson et al. [54], researchers made a very comprehensive review with a dataset that lists manufacturer, model, size, minimal intercondylar notch distance, and position of different femoral components. This is of practical use when planning the operative management of periprosthetic supracondylar femoral fractures regarding different femoral prosthesis and whether we should consider intramedullary nailing. An additional article worth mentioning is that which was written by Bobak et al. [55], in which researchers discuss a salvage technique, wherein they injected cement in a well-aligned femur and then inserted a retrograde nail without pressurization of the cement. It was accomplished in five octogenarian female patients with Rorabeck Type II fractures, who had advanced osteoporosis, and were followed for a median time of 12 months after surgery. Postoperatively, the patients began their mobilization on the first day with gentle continuous passive movement exercises. Toe-touch weight-bearing was initiated within 48 h post-surgery using walking aids. The results presented indicated that the patients had a median postoperative Oxford Knee score of 34 (range 24–42) and a median quality of life Euro Quality of Life 5D score of 0.69. When comparing this score with the weighted health status of the general UK population sample for age and sex, the mean for octogenarian women was also found to have the exact same value of 0.69.

## External fixation

In 2010, Beris et al. [56] published an article presenting three cases of periprosthetic fractures following total knee replacement treated with the Ilizarov external fixator, each of which had a follow-up of at least 3 years. The first patient had a Rorabeck type II fracture. Thirty-six months after injury, the patient’s left lower extremity showed satisfactory alignment and preservation of the joint congruity. In addition, the patient was mobilizing independently. The second patient had sustained a Rorabeck type I fracture. The device was removed at 6 months, at which time, the patient’s right lower extremity showed an excellent alignment. At follow-up, 42 months after injury, the knee range of motion was as it was prior to the operation. The third patient had sustained a Rorabeck type II fracture. Thirty-six months postoperatively, the tibiofemoral alignment was almost anatomical, and knee range of motion was comparable to pre-fracture status (0°–100°).

Another study was published by Refaat et al. [57], in 2015, which presents the case of a 54-year-old woman with a Rorabeck type II fracture. She was treated with a uniplanar external fixator. Three months following surgery, she was tolerating weight-bearing on the injured extremity with no pain. At 6 months, knee radiographs showed fracture consolidation with active range of motion of 10° to 120° and no pain.

The last case we would like to present was written by Assayag et al. [58] in 2018. The writer describes a simple and effective surgical technique using circular hexapod external fixation in two patients with poor soft-tissue envelope. This accompanied a periprosthetic tibia fracture around a well-fixed knee arthroplasty, where the tibial stem leaves little room for screw fixation. The first case is of a 48-year-old female who sustained a right periprosthetic Felix type 2A tibial fracture. CHEF (circular hexapod external fixation) was elected as a method of choice to obtain rigid fixation and provide accurate reduction. Contact between the external fixation pins and the prosthesis was avoided. Using a web-based software program, a gradual reduction in all planes was achieved. At follow-up 18 months after the injury was sustained, the patient was mobilizing independently, with a knee range of motion of 0°–120° and had resumed her pre-fracture level of activity. The second case is of a 78-year-old woman who sustained a left closed periprosthetic proximal tibia and fibula fracture around a well-fixed TKA implant. She was treated with CHEF and gradual reduction of the fracture. Within 2 weeks, she progressed to full weight-bearing. At the 7-month follow-up, she was ambulating with a walker and a drop lock-hinged knee brace for her long-standing quadriceps insufficiency.

To conclude this section, we would like to point out that when surgical intervention is selected to be the best option, the operation that one should select is the one that offers the best possible stability and will cause the least surgical trauma. As we saw with the presented cases, if the prosthesis is stable, external fixation is a reasonable option due to the fact that it complies with the prerequisites of quick, atraumatic surgery while simultaneously providing adequate stabilization. The device is applied percutaneously, causes little damage to the soft-tissue envelope, preserves the fracture hematoma, and does not require bone grafting. As the construct is stable enough to allow loading, early mobilization begins, and this permits quick rehabilitation and an early complication-free discharge.

## Revision total knee replacement

Multiple strategies were described in this review when dealing with a periprosthetic fracture around the knee. All of those methods aimed to achieve a functional, well-aligned, and stable knee, with minimal damage to the surrounding tissue. The common treatments are either osteosynthesis with a retrograde intramedullary nail or fixed-angle plate fixation. However, when dealing with a fracture with a loose or misaligned prosthesis, or one close to the joint, merely fixating the bone is usually not enough to fully heal the patient. Overall, when discussing the option of revision TKA for periprosthetic fractures, there are only a few studies that exist. Those studies are all small case series on fractures involving very distal fractures or fractures with loose implants.

In an article by Saidi et al. [59], researchers reviewed 23 patients with an average age of 80 years who suffered from comminuted distal periprosthetic femur fractures. They separated the patients into three groups which included the following: allograft prosthesis composite, distal femur replacement prosthesis (endoprosthesis), and conventional revision system. Although the amount of people who participated in this study was small, all three groups had similar functional outcomes. The distal femoral replacement group did not have an increased complication rate; on the contrary, the recovery of patients was quicker with a shortened surgical time and decreased blood loss. Thakur et al. [60] reported the results in 16 knees with acute Su type III, supracondylar periprosthetic fractures. All patients mentioned in their article returned to pre-fracture activity level. Additionally, all patients had a full union with the use of cemented constrained revision TKA implants.

In conclusion, if the bone stock is adequate, fracture reduction and a stemmed revision arthroplasty are a functional option. We should consider this option when the ligamentous structures provide adequate stability and there is an adequate bone stock after primary prosthesis removal [31, 61]. When we encounter fractures that involve bone stock deficiency, the choices that we have are allograft prosthesis composite or distal femur replacement endoprosthesis.

## Endoprosthesis

As in the previous section, when discussing a fracture that causes instability of the prosthesis, we must change the prosthetic component together with fixation of the bone component. But what if the fracture is so severe or the bone is so weak that we have to deal with three components simultaneously: bone fracture, unstable prosthesis, and loss of bone? We must use distal femoral replacement prosthesis (endoprosthesis). This allows us to use augments that will cover for bone loss. In this section, we will answer the question of whether that method allows our patient to live a normal life after the operation, or if we should look for different methods for this type of injury.

We will address two articles, each of which found reasonable results in their studies. In an article by Jassim et al. [62], the author presented a study that involved 11 patients with a mean age of 81 years. The patients suffered from periprosthetic fractures that had an unstable prosthesis with poor bone stock. Each participant was closely followed for 33 months. The results indicated that all implants survived without the need for re-operating, and the patients had few complications and acceptable functional outcomes. Another article that was previously discussed above, by Saidi et al. [59], reviewed seven distal femoral comminuted periprosthetic fracture patients who were treated with endoprosthesis, and compared the surgical complication and functional status to two other operational methods. They concluded that distal femur endoprosthesis, when performed by experienced hands, should be considered in patients with advanced age and poor bone quality who require early mobilization. In conclusion, we can state that this procedure is a viable option for patients suffering from complex fractures with massive bone loss who have no other treatment option. However, this procedure requires highly experienced orthopedic surgeons who are familiar with this procedure and special arthroplasty equipment and implants.

## Conclusion

A well-aligned and mobile knee joint in combination with a painless and unassisted, fully ambulatory patient should be a treatment goal. We propose that the decision regarding treatment options should be a team decision, which involves a highly qualified orthopedic surgeon, an anesthesiologist, an internal medicine specialist and, most importantly, the patient. In patients with stable fractures or those who are not eligible for surgery due to medical comorbidities, conservative non-surgical methods can be used that yield acceptable results. In patients who suffer from stable or unstable fractures, but possess good bone stock and stable prosthesis, the choices include both locking plate and intramedullary nailing, although, as we saw, external fixation is a method that allows our patients early ambulation with preservation of soft tissue while showing good results. Therefore, this method should be considered in this type of fracture. Fractures that have unstable prosthetic components but a good bone stock should be treated with revision surgery. In our experience as an orthopedic department in a tertiary facility, those are the majority of patients. It is our belief that the best way to treat these kind of fractures is with revision arthroplasty, when the bone stock is adequate and the ligamentous structures provide adequate stability, while those fractures that suffer from poor bone stock and unstable prosthesis should be treated with endoprosthesis. Another important issue we would like to point out is the solidity in recognizing the true severity of the periprosthetic fractures. It is not uncommon that a senior surgeon decides the best operative method only after the fracture is seen in the operating room, despite all the classification available.
